# Genomic and Effector‐Based Insights Into *Austropuccinia psidii*–Host Interactions Informing RNAi and Resistance Development

**DOI:** 10.1111/mpp.70190

**Published:** 2026-02-05

**Authors:** Jovarn V. Sullivan, Sophie E. Eccersall, Grant R. Smith, Renwick C. J. Dobson, Claudia‐Nicole Meisrimler

**Affiliations:** ^1^ Biomolecular Interaction Centre, School of Biological Sciences University of Canterbury Christchurch New Zealand; ^2^ New Zealand Institute for Bioeconomy Science Limited Lincoln New Zealand; ^3^ Department of Biochemistry and Pharmacology, Bio21 Molecular Science and Biotechnology Institute University of Melbourne Parkville Victoria Australia; ^4^ Riddet Institute, MacDiarmid Institute for Advanced Materials and Nanotechnology University of Canterbury Christchurch New Zealand

**Keywords:** *Austropuccinia psidii*, effector proteins, infection mechanism, Myrtaceae, myrtle rust, phytopathology, rust fungi

## Abstract

*Austropuccinia psidii* is a biotrophic basidiomycete and the causal pathogen of myrtle rust. The pandemic biotype infects over 480 Myrtaceae species and has caused functional extinction of myrtaceous species on the east coast of Australia, threatening numerous others worldwide. In planta resistance has been extensively explored, and resistant phenotypes are used in breeding programmes. At a molecular level, loci conferring resistance and secondary metabolite pathways activated during infection are being defined. A key component necessary to investigate this plant–pathogen interaction is an assembled and annotated pathogen genome. The *A. psidii* genome, determined to be one of the largest fungal genomes assembled to date, has a haploid size of 1 gigabase. Many putative effector sequences are present in the *A. psidii* genome: effectors are relatively small proteins that have been shown in other pathogen–host systems to facilitate infection through manipulation of the host's cellular processes. Some *A. psidii* effectors are expressed early during urediniospore germination and initial invasion of plant tissues, and thus may be unique targets for pathogen control. For example, in vitro RNA interference (RNAi) targeting the expression of *A. psidii* effector proteins for disease control has been demonstrated in laboratory and green/glasshouse experiments, but has yet to be tested in situ. Emerging host genomes and the characterisation of *A. psidii* effectors will continue to shed light on *A. psidii*–host interactions, aiding in the creation or optimisation of new treatments. Alternatively, treatments such as nanobodies or synthetic decoy resistance proteins could provide new means of disease prevention.

## Graphical abstract

Genomic and effector‐based insights into *Austropuccinia psidii—*host interactions are informing RNAi treatments and aiding resistance development for myrtle rust disease.

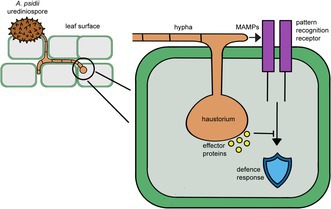

## 1 Introduction

*Austropuccinia psidii* (formerly *Puccinia psidii* (Beenken 2017)) is the causal pathogen of myrtle rust disease. Myrtle rust is characterised by bright yellow‐orange coloured urediniospores that extrude from uredinia on new plant growth, including young leaves, stems and flowers (Beresford et al. 2020; Fensham et al. 2020). Myrtle rust poses a significant threat to native and productive estates, infecting and ultimately killing plants of cultural, ecological and economic importance.

Typically, rust fungi have narrow primary host ranges, often with only one primary host (Aime et al. 2017). The pandemic biotype of *A. psidii* infects over 480 species in the Myrtaceae family (Soewarto et al. 2019), which is one of the world's largest angiosperm families, with over 5000 species, primarily native to the Southern Hemisphere (Thornhill et al. 2015). A wide host range is not typical for obligate biotrophic pathogens, as interactions between the pathogen and host plant are a result of coevolution, resulting in a narrow host range (Aime et al. 2017; McDowell 2011). The pandemic biotype of *A. psidii* challenges the ‘evolutionary arms race’ paradigm and models such as Flor's gene‐for‐gene hypothesis (Flor 1942), proposed over 80 years ago, and the Red Queen hypothesis, proposed by Van Valen over 40 years ago (Van Valen 1973). These hypotheses suggest that changes in plant defences drive the selection of pathogen infection mechanisms and vice versa, where plants evolve resistance (R) genes to overcome pathogens and pathogens evolve avirulence (avr) genes to overcome plant defences (Jones and Dangl 2006; Kazan and Lyons 2014). The pandemic biotype of *A. psidii* challenges these hypotheses by showing uniform infection dynamics across species within its host range that are separated by 65 million years of evolution (Luo et al. 2025).

Another unusual aspect of *A. psidii* is its extremely large geographical range. *A. psidii* was first described on *Psidium guajava* (=*Psidium pomiferum*) in South America in 1884 (Winter 1884). Since then, several biotypes have been described and categorised as genetically distinct clusters linked by host range and geographic regions (Stewart et al. 2018). Most described biotypes are restricted to South America; however, the South African (Roux et al. 2013) and the ‘pandemic biotype’ are widespread beyond this region. The pandemic biotype is the most prevalent, currently reported in South, Central and North America, Asia and Oceania (Granados et al. 2017; du Plessis et al. 2019; Stewart et al. 2018) and is genotypically distinguishable from the other *A. psidii* biotypes. The pandemic biotype is defined by two closely related *A. psidii* clusters with unique multilocus genotypes that are widely distributed across a diverse host range (Stewart et al. 2018). They are genetically distinct from those clusters found on specific hosts in a limited range and also distinct from the unique multihost South African strain (Roux et al. 2016).

**FIGURE 1:**
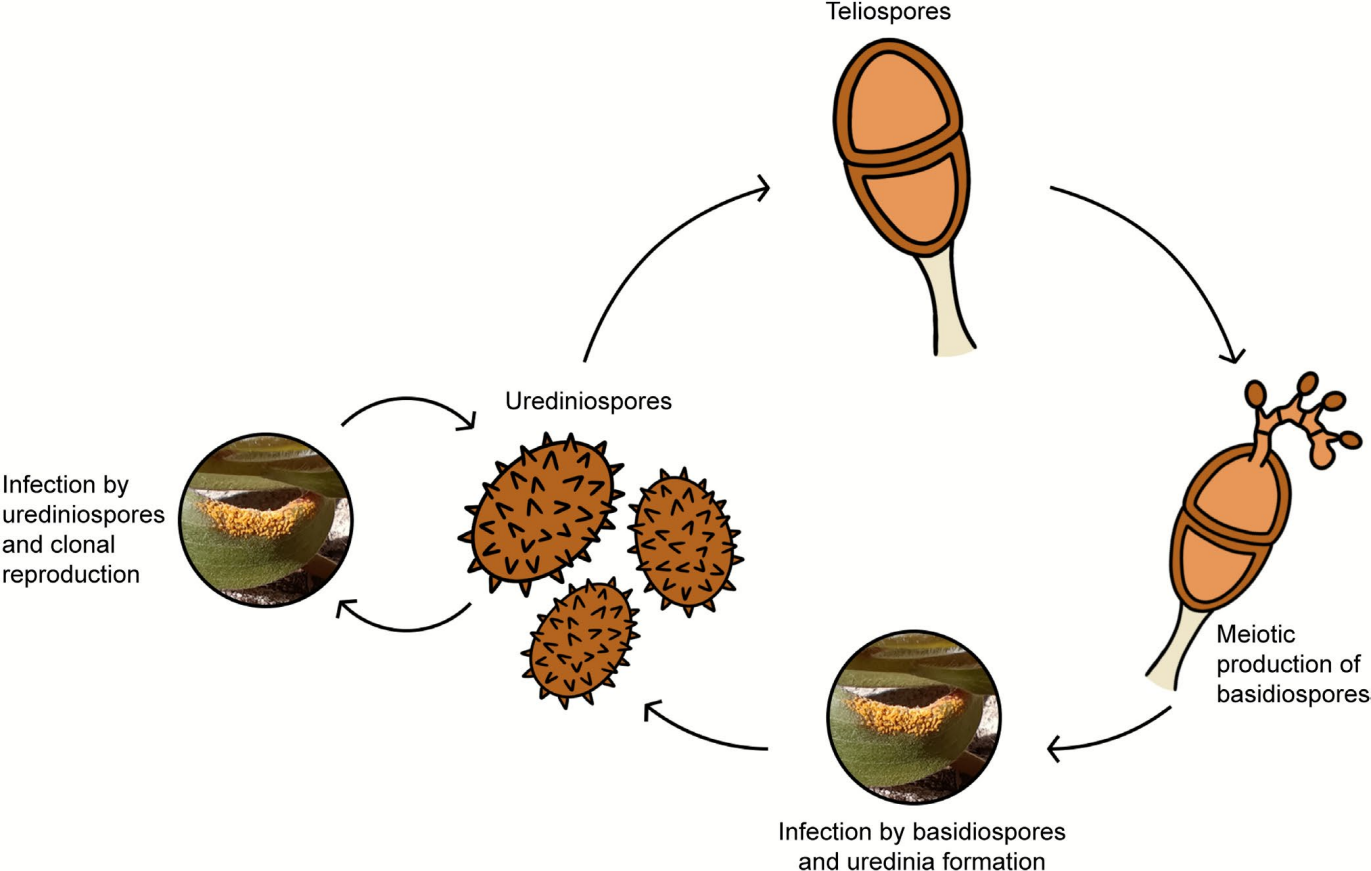
Life cycle of *Austropuccinia psidii*. Urediniospores infect hosts and reproduce asexually, producing clonal urediniospores that then repeat the cycle. Teliospores are sometimes formed from urediniospore infection. Teliospores are a diploid two‐cell spore stage that, through sexual recombination, produce basidiospores. Basidiospores then infect hosts and form uredinia containing recombinant urediniospores.

Three *A. psidii* life stages are observed in nature, distinguishable by spore type: urediniospore, teliospore and basidiospore. The asexual dikaryotic urediniospore stage has two separate haploid nuclei (Coutinho et al. 1998; Tobias et al. 2020) that do not exchange genetic material, so mutation is the principal mechanism responsible for genetic change. This results in high levels of heterozygosity and, therefore, multiple gene variants (haplotypes) between the two nuclei (Edwards et al. 2022; Schwessinger et al. 2020). The most abundant spore type is the urediniospore, and dispersion of *A. psidii* is primarily by windborne urediniospores (McTaggart et al. 2018). The diploid two‐cell teliospore stage meiotically produces the final basidiospore stage, which can have either one or two nuclei (Morin et al. 2014). Basidiospores then infect a host and produce recombinant urediniospores, which can infect hosts and either clonally reproduce or form teliospores (Figure 1). In contrast to the urediniospores, teliospores and basidiospores are relatively rare in the wild (Glen et al. 2007; Morin et al. 2014). The sexual stages have been observed in the invasive populations of *A. psidii*, with the long‐term advantages of sexual reproduction likely driving rapid adaptation to new environments associated with its wide geographic spread (McTaggart et al. 2020).

Current control of *A. psidii* is achieved using fungicides (Beresford 2025; Carnegie and Pegg 2018; Pathan et al. 2020) and breeding programmes using phenotypically resistant plants (Almeida et al. 2021; Junghans, Alfenas, Brommonschenkel, et al. 2003; Junghans, Alfenas, and Maffia 2003; Yong et al. 2021). The original fungicide treatments of demethylation inhibitors mixed with triazole fungicides are effective for short‐term control, but an optimal application time is required for control (Pathan et al. 2020). However, excessive use of fungicides can lead to the development of resistance through mutation (McDonald et al. 2019). With no proven control treatment at present, fungicide treatment is used in conjunction with monitoring the spread of the fungus, particularly in Australia and Aotearoa‐New Zealand (Carnegie and Pegg 2018; Diprose et al. 2022). Unfortunately, no long‐term solution to suppress infection or spread has been identified.

Like other plant pathogens, *A. psidii* evolves mechanisms to evade or suppress the host plant's defence responses. Plant pathogens produce specialised protein virulence factors called effectors that are secreted to disrupt and manipulate the host's defences (Jones and Dangl 2006). The effector protein repertoires of a range of plant pathogens have been investigated, including several *Phytophthora* species (Cox et al. 2022; Engelbrecht et al. 2021; Haas et al. 2009; McGowan et al. 2020), *Pseudomonas syringae* (Dillon et al. 2019), *Parastagonospora nodorum* (Jones et al. 2024), *Magnaporthe oryzae* (Devanna et al. 2022), *Ralstonia solanacearum* (Landry et al. 2020; Mukaihara et al. 2010), *Melampsora lini* (Nemri et al. 2014) and *Fusarium oxysporum* ff. spp. (van Dam et al. 2016).

**FIGURE 2:**
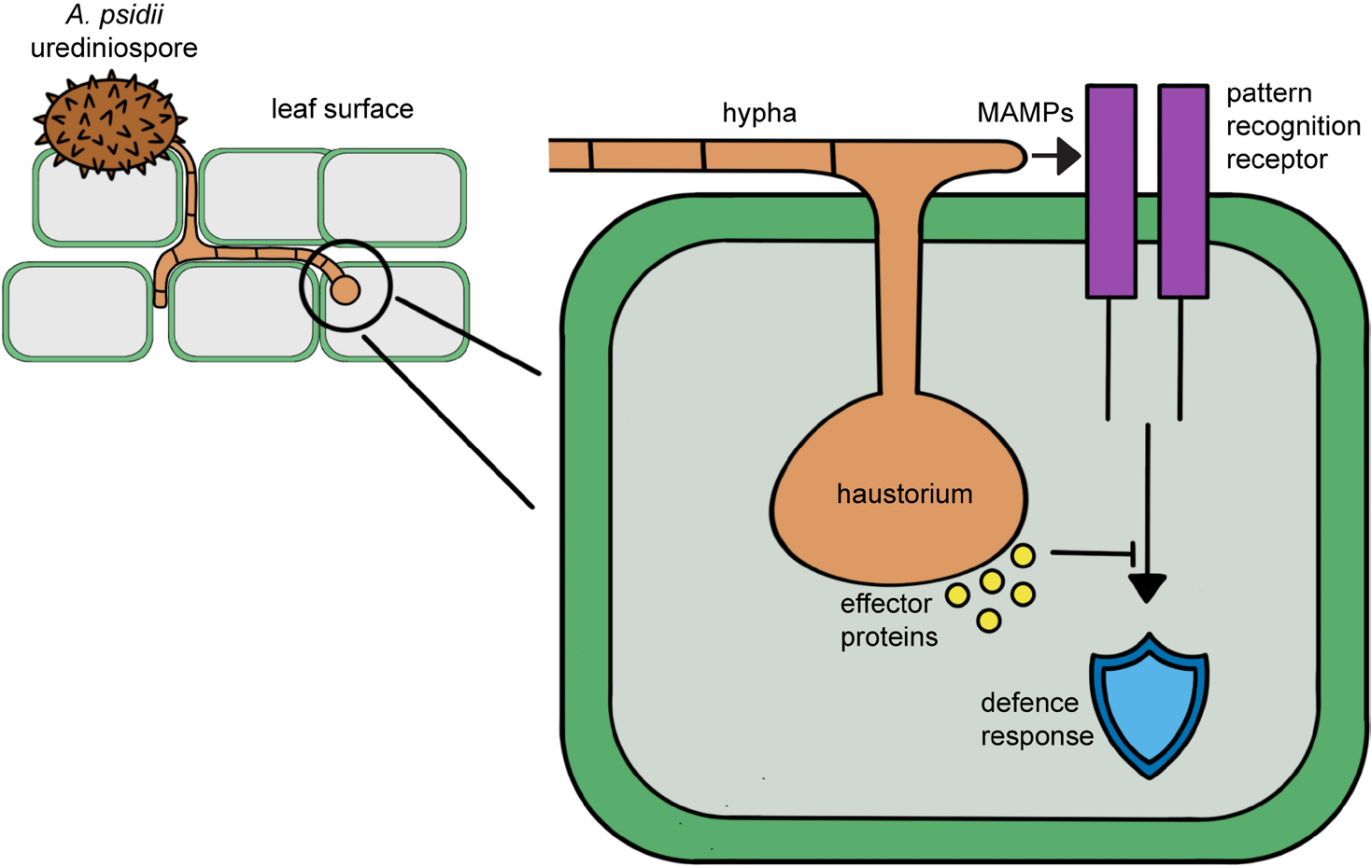
Schematic of *Austropuccinia psidii* infection and usage of effectors. *A. psidii* urediniospores germinate and penetrate the leaf epidermis. After penetration, the haustorium forms within the mesophyll cells. Basal plant defences recognise common microbe‐associated molecular patterns (MAMPs), such as fungal cell wall chitin, via their pattern recognition receptors, initiating a defence response. To combat this defence response, *A. psidii* secretes cytoplasmic effector proteins to directly manipulate and interact with the host's cellular processes.

Effector proteins are often a research focus when investigating mechanisms of infection by plant pathogens. There are two types of effector proteins: apoplastic effectors that operate in the extracellular spaces, including protease inhibitors, peroxidase inhibitors, chitin‐binding proteins and hydrolytic enzymes (Giraldo and Valent 2013; Haas et al. 2009); and cytoplasmic effectors that operate within the plant cell and directly manipulate and interact with host cellular processes. Cytoplasmic effectors are directly secreted from the haustorium, a specialist infection structure, into the plant cell (Giraldo and Valent 2013) (Figure 2). Many effectors have conserved amino acid motifs that have evolved during the ‘molecular arms race’ (Wang, Shakoor, et al. 2021) between the pathogen and its host. These conserved motifs have distinct roles in the infection process and have been extensively researched and reviewed over the past two decades (Liu et al. 2019).

As an emerging pathogen, we have only recently turned our attention to the *A. psidii* effector repertoire and its functions. Here, we review the basis of in planta resistance to *A. psidii*, the assembly of the pandemic biotype genome, identifying effectors from fungal transcripts during infection and emerging effector‐based treatments using RNA interference (RNAi). Because myrtle rust is now a widespread disease, key questions include how *A. psidii* infects such a wide host range across a broad geographical range, and the molecular basis of the *A. psidii*–host infection process. The answers to these questions will contribute to the development of effective control strategies.

## 2 Exploiting In Planta Resistance for Preventing *A. psidii* Infections

Although fungicide treatments, such as tebuconazole combined with trifloxystrobin, or cyproconazole combined with azoxystrobin, reduce the symptoms of infection on already infected plants, they have limited efficacy to limit the spread of the pathogen (Adusei‐Fosu et al. 2021; Pathan et al. 2020), highlighting the need for alternative solutions. Consequently, the search for alternative treatments has focused on exploring in planta resistance. As part of the search for *A. psidii* resistance, significant effort has been made to identify species that exhibit phenotypic resistance. A modified Junghans scale (Junghans, Alfenas, Brommonschenkel, et al. 2003; Junghans, Alfenas, and Maffia 2003) has been used to define levels of phenotypic resistance exhibited in some species of Myrtaceae (Pegg et al. 2014; Smith et al. 2020) (Table 1). A similar set of classes has also been developed to classify stem infection phenotypes (Smith et al. 2020). Hypersensitive response and associated limited necrosis observed in plants, rated 2 on the modified scale, ultimately limit the growth of the pathogen throughout the plant tissue. Plants that are susceptible to *A. psidii* fall into ratings 3–5. This may indicate they lack the genetic repertoire of resistance (R) genes needed to recognise the corresponding avirulence (avr) genes from *A. psidii*. Susceptible plants when infected show typical symptoms and develop pustules, characteristic of myrtle rust disease (Yong et al. 2019). Myrtaceous species exhibiting some level of phenotypic resistance to *A. psidii*, such as *Eucalyptus grandis* (Junghans, Alfenas, Brommonschenkel, et al. 2003; Junghans, Alfenas, and Maffia 2003), *Eucalyptus obliqua* (Yong et al. 2019), *Eucalyptus globulus* (Butler et al. 2016) and *Eucalyptus pellita* (Santos et al. 2014), have been used in breeding programmes to exploit the genes conferring resistance. Eucalypts containing the *A. psidii* resistance loci *Ppr1–Ppr5* (*Puccinia psidii* resistance) have been grown in Brasilian eucalypt plantations for nearly 20 years (Almeida et al. 2021; Junghans, Alfenas, Brommonschenkel, et al. 2003; Junghans, Alfenas, and Maffia 2003; Yong et al. 2021). The locus *Ppr1* was first identified in *E. grandis* (Junghans, Alfenas, Brommonschenkel, et al. 2003; Junghans, Alfenas, and Maffia 2003), with the loci *Ppr2–Ppr5* being identified later in *E. globulus* (Butler et al. 2016). Identifying these plant loci that convey resistance to *A. psidii* led to hybridisation and molecular breeding to produce myrtle rust‐resistant eucalypts (Almeida et al. 2021; Alves et al. 2012; Yong et al. 2021). The *Ppr* loci have not yet been reported in other genera of Myrtaceae. This is likely due to the myrtle rust disease being a pathosystem that has not co‐evolved in novel hosts. A new *A. psidii* biotype in Brasil has overcome the resistance of the widely planted hybrid of *E. grandis* and *E. urophylla* (Almeida et al. 2021), with *Eucalyptus* breeding programmes using resistance phenotyping to select for resistant clones for widespread planting. In earlier breeding programmes, resistance was selected at the final stage. However, selecting for resistance at the initial stage was found not to affect plant height, density or annual growth rate (Arriel et al. 2024).

**TABLE 1:** Modified Junghans scale (Junghans, Alfenas, Brommonschenkel, et al. 2003; Junghans, Alfenas, and Maffia 2003) from Pegg et al. (2014) and Smith et al. (2020) for defining levels of phenotypic resistance against *Austropuccinia psidii* by visual assessment.

Rating	Leaf visual symptoms
1	No symptoms or presence of yellow‐flecking
2	Presence of hypersensitive response, with flecking or necrosis
3	Small pustules that are < 0.8 mm in diameter, with one or two uredinia
4	Medium‐sized pustules that are between 0.8 and 1.6 mm in diameter, with several uredinia
5	Large pustules that are > 1.6 mm in diameter, with more than 20 uredinia on leaves, shoots or petioles

The mechanistic basis of this resistance has been investigated in *E. grandis* . Interestingly, resistant individuals (rating 1) show a significantly increased expression of brassinosteroid‐mediated signalling genes when compared to susceptible individuals (rating 5) (Swanepoel et al. 2021). These genes, located at the *Ppr3* and *Ppr5* loci, were identified through a transcriptomic approach, revealing a secondary metabolite‐based resistance mechanism using brassinosteroids as well as up‐regulation of salicylic acid, jasmonic acid and ethylene pathways (Swanepoel et al. 2021). As phenotypically resistant individuals contain these *Ppr* loci conferring secondary metabolite resistance, they are prime candidates for breeding programmes such as those in Brasil. Other studies have also confirmed the involvement of secondary metabolites in the resistance to *A. psidii* through the means of metabolomic studies focusing on infected *Melaleuca quinquenervia* plants. Flavonoids and organoheterocyclic compounds are heightened in resistant individuals (rating 1) when compared to susceptible plants (rating 5), with flavonoids being enriched in the leaves of resistant plants pre‐infection and organoheterocyclic compounds being enriched in resistant individuals independent of time post‐infection (Moffitt et al. 2022).

Resistance to *A. psidii* has also been identified in novel myrtaceous host species outside Brasil. Threatened Australian rainforest natives, *Rhodamnia rubescens* and *Rhodomyrtus psidioides*, were tested for phenotypic resistance to *A. psidii* via urediniospore inoculation. No resistance was observed in *R. psidioides*, but some *R. rubescens* individuals showed resistant phenotypes (Chen et al. 2025). Similarly, phenotypic assays in Aotearoa‐New Zealand reveal varying levels of resistance in native species *Leptospermum scoparium* , *Kunzea robusta* and *Kunzea linearis* (Smith et al. 2020). Notably, *L. scoparium* displays three forms of resistance: a putative immune response, a hypersensitive leaf response and a putative stem‐based resistance. The same study demonstrated that another Aotearoa‐New Zealand native, *Metrosideros excelsa* has very little resistance, while *Lophomyrtus bullata* and *Lophomyrtus obcordata* have no resistance at all (Smith et al. 2020), with localised extinctions predicted for all three (McCarthy et al. 2024). Although no species is fully resistant to *A. psidii*, individual plants within highly susceptible species can show resistance. This highlights the potential for informed breeding and hybridisation strategies to restore vulnerable populations by selecting and propagating resistant individuals.

In summary, while breeding and hybridisation offer an effective long‐term solution by enhancing population‐level resistance, these approaches will take years to implement. Meanwhile, the pandemic variant of *A. psidii* is causing localised extinctions (Fensham et al. 2020) and is likely to cause more (Sutherland et al. 2020), underscoring the urgent need for complementary intervention strategies.

## 3 Assembling the Genome of *A. psidii* Revealed a Large Number of Effectors

Developing new control options that prevent or cure *A. psidii* infection requires a detailed understanding of the interaction with the plant host. The first assembly of a highly contiguous reference genome for the *A. psidii* pandemic biotype by Tobias et al. (2020) established that *A. psidii* has an estimated haploid size of one gigabase (1 Gb), the largest assembled fungal genome to date. Analysis of this genome revealed a proteome of ~19,000 and ~16,000 protein‐coding genes in the primary and secondary assemblies, respectively, consistent with rust fungi in terms of gene count. However, *A. psidii* displays markedly expanded intergenic regions compared to other species in the Pucciniales, a result of transposable element insertions, while gene lengths including untranslated regions (UTRs) remain comparable with other rust genomes. A total of 367 and 304 putative effector genes were identified in the primary and secondary assemblies, respectively. These effectors are not spatially compartmentalised and exhibit similarly extended intergenic distances found in other gene sets. Apoplastic localisation, a hallmark of rust pathogen effectors, was predicted for 47% and 40% of effectors in the respective assemblies, supporting a non‐cytoplasmic mode of host manipulation. In planta RNA‐seq mapping confirmed expression for thousands of predicted genes, including 78 and 63 effectors, further supporting their potential functional roles during infection. Overall, these findings are consistent with a rust‐like genomic architecture in *A. psidii*, with expanded intergenic regions and a substantial apoplastic effector repertoire that likely contributes to its pathogenic success (Tobias et al. 2020).

Further research on the *A. psidii* genome includes the first chromosome‐level phased genome for the pandemic biotype of *A. psidii* (Edwards et al. 2022). Phased assembly of the genome produced two assemblies that are both ~1 Gb, labelled haplotype 1 and haplotype 2, consistent with the total genome size from the first assembly. This version of the genome was slightly improved in BUSCO completeness, increasing from 91.4% in the first genome to 92.0%. Each haplotype contained 18 chromosomes, consistent with other rust fungi. Over 1200 putative effector genes were identified in these assemblies and mapped to the two haploid genomes: 617 to haplotype 1 and 616 to haplotype 2. Of these putative effector genes, 595 are shared homologues present in both haplotypes, with chromosome 3 showing an abundance of predicted effector genes, 68 and 67, respectively, for the two haplotypes. Of those predicted effector genes not shared between the haplotypes, 22 are only found in haplotype 1 and 21 are only found in haplotype 2, suggesting high levels of heterozygosity. It is also noted that many of the effector genes are close to the telomeric regions of the chromosomes, a region known for diversity in fungal effectors (Gan et al. 2021). With the characterisation of haplotypes, studies focusing on the heterozygosity of *A. psidii* are now possible, including how this contributes to pathogenesis and the wide geographical and host range.

A third version of the genome has recently been assembled and analysed (Luo et al. 2025). This version builds upon and improves the previous two, which used early‐generation sequencers and long‐read assembly software, resulting in residual errors, especially in repeat‐rich regions. To overcome the large size and repetitive regions of the genome, a combination of PacBio HiFi, Oxford Nanopore Technologies ultralong reads and Hi‐C data sequencing technologies was used and assembled using Hifiasm software. This led to the assembly having a BUSCO completeness of 92.0%, the same as the second version and slightly higher than the 91.4% of the first genome. This also led to the production of a telomere‐to‐telomere genome for the pandemic biotype of *A. psidii*, with two assemblies of ~1 Gb named haplotype A and haplotype B. These assemblies comprise 18 haploid chromosomes, consistent with other rust fungi. However, there is an unequal number of chromosomes in each haplotype, with 19 chromosomes in haplotype A and 17 in haplotype B. It is proposed that this is a result of chromosome 14B being relocated from the haplotype B nucleus to the haplotype A nucleus. Interestingly, chromosome 14B is stably inherited in urediniospores, with chromosome 14 showing greater homology than other chromosomes, as mutations within these chromosomes are likely repaired through homologous recombination (Luo et al. 2025; Moynahan and Jasin 2010). In contrast, the other chromosomes are more likely to diverge because of mutation, leading to the observed high levels of heterozygosity. The transposable element insertions reported in the first version genome were confirmed in the third version genome, with 89% of the genome composed of repetitive regions, which are subsequently silenced by DNA methylation (Luo et al. 2025; Zhou et al. 2020). In planta RNA‐seq mapping in *Syzygium jambos* confirmed expression for thousands of predicted genes, including 283 and 240 predicted effectors (Luo et al. 2025). Of the expressed effectors, 30 and 25 are found only in their respective haplotypes, the remainder having alleles in both haplotypes. Only 32 are consistently expressed, with allele‐specific expression occurring for most effectors (Luo et al. 2025). Refining of the *A. psidii* genome confirms expanded intergenic regions and unveils allele‐specific effector expression, potentially allowing for adaptive infection of hosts, which may be instrumental to its virulence.

## 4 Identifying *A. psidii* Effectors That Are Essential for Infection

Fungal transcript analysis from inoculated *E. grandis* by Swanepoel et al. (2023) identified 890 expressed genes, 43 of which are candidate effectors. Susceptible (rating 5) and resistant (rating 1) phenotype *E. grandis* plants were inoculated with *A. psidii* urediniospores and sampled at 12, 24 and 48 h and 5 days post‐inoculation. Samples were collected for RNA sequencing and were mapped to the *A. psidii* genome using the first version genome (Tobias et al. 2020) as a reference. Gene expression across the sampled time points shows that during the first 24–48 h, expression levels between the two plant phenotypes are somewhat comparable. However, after 48 h, *A. psidii* gene expression in resistant phenotypes dropped drastically, while expression in susceptible plants remained stable, with expressed genes increasing further 5 days post‐inoculation. Of the overall top 10 expressed genes, seven are shared between the two phenotypes, three of which are candidate effector proteins. In the susceptible phenotype, several unique virulence factors, including seven effectors, were identified, suggesting they might play a role in the successful infection of susceptible plants. To further investigate the potential virulence of the 43 candidate effectors, sequence comparisons using the Basic Local Alignment Search Tool (BLAST) showed that only 17 had homology with sequences from other organisms. Notable homologous sequences included a rust transferred protein, a hydrolase 76 protein and a small subunit ribosomal protein. Most putative effectors are uncharacterised or hypothetical proteins, highlighting the need for further investigation on their functions.

Similarly, fungal transcript analysis using susceptible (rating 5), hypersensitive resistant (rating 2) and resistant (rating 1) phenotype *L. scoparium* at 24‐ and 48‐h post‐infection by Frampton et al. (2024) revealed differential gene expression between the time points. Principal component analysis of genes expressed 24 h post‐inoculation found that they were highly similar compared to the genes expressed 48 h post‐infection, though both time points formed distinct clusters. Differential gene expression analyses across the time points found 16 predicted effector protein transcripts that are more abundant at 24 h than at 48 h post‐inoculation. Among these, three were in the top 10 differentially expressed genes: two have homologues in other *Puccinia* species, while the other one appears to be unique to *A. psidii*. There is a positive correlation between the expression of these three putative effectors. The drop in abundance of transcripts between 24 and 48 h suggests they are important during the early stages of infection of the plant. There is no functional annotation for any of the three effector genes, though they are being investigated, with initial production in *Escherichia coli* demonstrated (Sullivan et al. 2026). Interestingly, the top 10 differentially expressed genes found during the infection of *L. scoparium* (Frampton et al. 2024) are not found in the top 100 differentially expressed genes found during the infection of *E. grandis* (Hayashibara et al. 2023).

Hayashibara et al. (2023) investigated the *A. psidii* MF‐1 biotype, an *E. grandis* ‐specific variant found in Brasil, identifying 255 putative effector genes predicted from two in silico pipelines from the corresponding *A. psidii* MF‐1 draft genome. Most of the putative effectors are predicted to be apoplastic, with only 12% predicted to be cytoplasmic. Functionally, 65% of the identified effector genes have no annotation in the MF‐1 genome, in contrast to 23% annotated as hypothetical proteins, and the remaining 11% annotated with a putative function. The majority of the proteins annotated with a putative function show homology to those in other rust species and include enzymes such as hydrolases, chitin deacetylases and lipases. Seven of the 255 effectors were selected for an in vitro expression study to determine if their expression is affected by leaf cuticular waxes. *A. psidii* MF‐1 urediniospores were cultured in the presence of cuticular wax of *E. grandis* (susceptible) and *E. urophylla* (resistant) and sampled at 0‐, 6‐, 12‐ and 24‐h post‐inoculation. Reverse transcription‐quantitative PCR analysis of samples revealed that the putative effectors Ap28303 and Ap12491 were upregulated in the presence of cuticular wax from susceptible *E. grandis* , Ap28303 at 6 h post‐inoculation and Ap12491 across all time points. The other effectors were either downregulated in the presence of cuticular waxes or showed no significant changes in expression when exposed to either cuticular wax.

Hayashibara et al. (2023) provided the first in planta subcellular localisation of an *A. psidii* effector. The effector Ap28303 was predicted to be cytoplasmic (operating within the plant cells) and specifically localise in the nucleus, and was described as a protease inhibitor I9 domain‐containing protein. *Agrobacterium tumefaciens* ‐mediated transient expression in *Nicotiana benthamiana* showed that Ap28303 did appear to localise in the nucleus. Localisation of a protease‐inhibiting effector to the nucleus is atypical, as most have been observed to localise in the cytoplasm (Pretsch et al. 2013; Qi et al. 2019). This result led to the hypothesis that Ap28303 may be involved in establishing compatible infection in a susceptible host by suppressing host proteases, or may have a dual function of targeting nuclear proteins such as transcription factors due to its localisation in the nucleus (Hayashibara et al. 2023). The authors do not imply that Ap28303 could target host nuclear proteases, which do exist in planta and include deubiquitinases, SUMO‐proteases and metacaspases (Doelling et al. 2007; Morrell and Sadanandom 2019; Murtas et al. 2003; Ruiz‐Solaní et al. 2023).

Many fungal pathogen effector proteins expressed during infection have been explored in detail, including effectors from *Colletotrichum higginsianum* (Robin et al. 2018), *M. oryzae* (Khang et al. 2010), *Melampsora larici‐populina* (Petre et al. 2015), *M. lini* (Dodds et al. 2004), and *Ustilago maydis* (Shi et al. 2023). Techniques such as co‐immunoprecipitation with mass spectrometry, Turbo‐ID and yeast‐two‐hybrid systems have been instrumental for characterising effector localisation and protein–protein interactions, providing important insights into the virulence strategies of fungal pathogens, including rust species. However, the specific roles of effectors during *A. psidii* infection remain to be fully resolved.

## 5 Potential Control of *A. psidii* Using RNAi

RNAi is a conserved, post‐transcriptional regulatory pathway in eukaryotes involving the processing of double‐stranded RNAs (dsRNAs) into small interfering RNAs (siRNAs) that guide the degradation of complementary target transcripts, thereby silencing gene expression (Mocellin and Provenzano 2004). Recent studies have demonstrated that rust fungi, including *A. psidii*, possess the core RNAi components—Dicer, Argonaute and RNA‐dependent RNA polymerase—indicating a functional RNAi system in these obligate biotrophs (Degnan, McTaggart, et al. 2023). For example, in *Puccinia graminis* f. sp. *tritici*, small RNA (sRNA) sequencing revealed two infection stage‐specific waves of sRNAs and tightly regulated expression of RNAi genes, suggesting a role in both gene regulation and transposable element silencing during infection and sporulation (Sperschneider et al. 2021). Importantly, recent research on the *A. psidii* genome and effector candidate identification (Frampton et al. 2024) led to a promising treatment option for myrtle rust using RNAi for the silencing of *A. psidii* genes (Degnan et al. 2024). The initiation of RNAi in plant pathogens can take two routes: host‐induced gene silencing (HIGS) and spray‐induced gene silencing (SIGS). HIGS is an existing defence process that occurs in plants, where a host plant uses RNA silencing to affect gene expression of pathogens; this process has been extensively reviewed (Baulcombe 2015). Introducing HIGS in plants is a transgenic approach, requiring transformation of the plant species. SIGS does not involve genetic modifications to induce RNAi and is delivered via exogenous application of dsRNAs that guide the targeting of one or more pathogen transcripts (Wang and Jin 2017). Various SIGSg approaches have been tested and demonstrated control of a range of plant pathogens. Application of dsRNA by SIGS has targeted both virulence‐related genes (Li et al. 2022; McRae et al. 2023; Hayashibara et al. 2023; Qiao et al. 2021; Sarkar and Roy‐Barman 2021) and non‐virulence‐related genes, such as those involved in respiration, glycosylation (Ruiz‐Jiménez et al. 2021) and chitin synthesis (Saito et al. 2022). Silencing virulence‐related genes in plant pathogens, such as effector proteins, represents a putatively unique target for the treatment and prevention of plant infection. dsRNA treatments including SIGS have been suggested to be more beneficial because the pathogens being targeted are unlikely to evolve resistance to dsRNA, in contrast to fungicide treatment, as resistance would require the loss of essential genes or the RNAi pathway (Degnan, McTaggart, et al. 2023).

Use of dsRNAs to control *A. psidii* was tested by Degnan, McTaggart, et al. (2023), using dsRNAs targeting essential *A. psidii* genes. These dsRNA molecules were exogenously applied to spores and found to associate externally or be internalised by urediniospores, but not by teliospores (Degnan, McTaggart, et al. 2023). During the urediniospore stage, targeting essential genes such as those critical for cellular function is more effective at preventing germination of spores than targeting genes that are preferentially transcribed in haustoria. This interaction is also found to be concentration‐dependent, with higher concentrations of dsRNA being more effective at inhibiting urediniospore germination (Degnan, McTaggart, et al. 2023). Application of dsRNAs that target the *β‐tubulin* (*β* *‐TUB*), *translation* *elongation factor 1α* (*EF1‐α*) and *ribosomal RNA 28S‐1* (*28S‐1*) RNAs led to withered germ tubes and germ tubes that did not produce appressoria, both on artificial surfaces and detached *S. jambos* leaves infected with *A. psidii* (Degnan, McTaggart, et al. 2023). In planta, application of these dsRNAs on 1‐year‐old *S. jambos* plants reduces disease incidence. Further dsRNA testing by Degnan, Shuey, et al. (2023) targeting *β‐TUB* and *EF1‐α* demonstrated prevention and cure of infection in planta at various stages of the *A. psidii* disease cycle. Importantly, the application of dsRNAs to *S. jambos* prevented infection when applied pre‐inoculation, reduced disease coverage when applied 6 days post‐infection, and improved plant health when applied 14 days post‐infection (Degnan, Shuey, et al. 2023). While targeting essential cellular genes reduced symptoms in *A. psidii*‐infected *S. jambos* (Degnan, McTaggart, et al. 2023; Degnan, Shuey, et al. 2023), silencing effector genes offers a distinct route for disease control.

Degnan et al. (2024), targeting the effector candidates identified by Frampton et al. (2024), applied dsRNAs against effector candidates EFC1, EFC2 and EFC3, which are expressed early in infection. All assay conditions had three biological replicates. Silencing EFC2 and EFC3 virtually abolishes germination of urediniospores in vitro (*p* = 0.0002 and *p* = 0.007, respectively); in planta, silencing EFC2 significantly reduces disease symptoms after 2 weeks (*p* = 0.019), while silencing EFC3 shows a similar trend but without statistical significance when compared to the controls (*p* = 0.072). EFC1 was discovered to be a gene family of secreted proteins with high intragenomic diversity, supported by a high rate of nonsynonymous (compared to synonymous) substitutions (dN/dS ratio) and significant likelihood ratio test values among discovered variants. In the phased assembly of the *A. psidii* pandemic biotype, three variants of EFC1 are found on chromosome 3A, the chromosome with an abundance of predicted effector proteins (Degnan et al. 2024; Edwards et al. 2022). Two EFC1 variants, EFC1‐01 and EFC1‐19, were targeted with dsRNA and exhibited distinct differences in their effect on urediniospore germination in vitro. While EFC1‐01 silencing did not change urediniospore germination rates, dsRNA targeting of EFC1‐19 significantly reduced germination (*p* = 0.0052) (Degnan et al. 2024). In planta application of dsRNA against EFC1‐01 and EFC1‐19 during *A. psidii* inoculation of *S. jambos* significantly inhibited infection and disease progression 2 weeks post‐inoculation (*p* = 0.0118 and *p* = 0.01104, respectively). However, when applied 3 days post‐inoculation, no curative effects were observed, suggesting that EFC1 is crucial in the early stages of *A. psidii* infection (Degnan et al. 2024).

Together these studies demonstrate that silencing putative effector proteins is an effective strategy for preventing and treating myrtle rust, justifying more work to evaluate its efficacy under field conditions.

## 6 Outlook and Future Perspectives

Studying effector proteins is critical for defining the infection mechanisms of plant pathogens. The early expression of effectors by *A. psidii* and the reduction of disease incidence when these putative effectors are silenced suggest that these genes are crucial for successful plant infection. Through the assembly of the *A. psidii* genome, the identification of putative effectors, and the demonstration of their importance for infection, some understanding of the basis of the extensive host range of the pandemic strain of *A. psidii* has been gained. Furthermore, recently assembled genomes of host species (Balkwill et al. 2024; Feng et al. 2021; Healey et al. 2021; Izuno et al. 2019; Myburg et al. 2014; Ouadi et al. 2023; Shen et al. 2023; Thrimawithana et al. 2019; Zheng et al. 2022) and new assemblies of the *A. psidii* genome (Luo et al. 2025) continue to provide further insights into this unique pathosystem, resulting in a deeper understanding of the *A. psidii*–host interaction.

Preliminary RNAi studies targeting *A. psidii* effectors in vitro suggest that this strategy may be a viable method to control myrtle rust disease, potentially replacing current fungicide treatments. The next step will be to transition this strategy from the lab to the field to test the efficacy of RNAi for myrtle rust control in situ. Alternative strategies for controlling *A. psidii* include designed nanobodies to specifically bind key *A. psidii* proteins, resulting in their functional neutralisation. The use of nanobodies has previously been reviewed for their potential in plant biotechnology (Wang, Yuan, et al. 2021) and could provide an alternative control and treatment option. A synthetic biology approach could also be applied against *A. psidii*, as engineered decoy resistance proteins have been shown to confer resistance to viral infection of *Arabidopsis* and soybean (Pottinger et al. 2020).

While targeting pathogen effectors is a control option, the molecular mechanisms underlying effector protein function are still unknown. How *A. psidii* effectors are delivered into host cells is not yet understood, though studies in other fungi (Oliveira‐Garcia et al. 2023; Zhang et al. 2024) provide models for future investigation. Where these effectors localise, and what they interact with, are still characteristics that remain unknown and are crucial for understanding the interactions between *A. psidii* and its hosts.

## Author Contributions

**Jovarn V. Sullivan:** writing – original draft preparation, writing – review and editing. **Sophie E. Eccersall:** writing – review and editing. **Grant R. Smith:** writing – review and editing. **Renwick C. J. Dobson:** writing – review and editing. **Claudia‐Nicole Meisrimler:** writing – review and editing.

## Funding

This work was supported by the Ministry for Business Innovation and Employment, 2223‐44‐005.

## Conflicts of Interest

The authors declare no conflicts of interest.

## Data Availability

The authors have nothing to report.
